# Effects of Guchang Capsule on Dextran Sulphate Sodium-Induced Experimental Ulcerative Colitis in Mice

**DOI:** 10.1155/2016/3150651

**Published:** 2016-05-24

**Authors:** Baoshan Liu, Tong Liu, Xiaohong Wang, Xin Zheng, Hong Wang, Lin Ma

**Affiliations:** ^1^Department of Traditional Chinese Medicine, Tianjin Medical University General Hospital, Tianjin 300052, China; ^2^Tianjin Zhongxin Pharmaceutical Group Corporation LTD., Tianjin 300457, China

## Abstract

Guchang capsule (GC) is a Chinese materia medica standardized product extracted from 15 Chinese traditional medical herbs and it has been clinically used in the treatment of intestinal disease. In this study, in order to extend the research of GC in intestinal disease, we were aiming to evaluate potential effects of GC on dextran sulphate sodium- (DSS-) induced murine experimental colitis and to elucidate the underlying mechanisms. GC treatment attenuated DSS-induced body weight loss and reduced the mortality. Moreover, GC treatment prevented DSS-induced colonic pathological damage; meanwhile it inhibited proinflammatory cytokines production in colon tissues.* In vitro*, GC significantly reduced LPS-induced proinflammatory cytokines production via inhibiting the activation of NF-*κ*B in macrophage cells, and the expressions of several long noncoding RNAs (lncRNAs) which were reported in regulating NF-*κ*B signaling pathway were obviously affected by adding GC into culture medium. In conclusion, our data suggested that administration of GC exhibits therapeutic effects on DSS-induced colitis partially through regulating the expression of NF-*κ*B related lncRNAs in infiltrating immune cells.

## 1. Introduction

Ulcerative colitis (UC) and Crohn's disease (CD), which are considered inflammatory bowel diseases (IBDs), are chronic gastrointestinal disorders characterized by inflammation in the intestine and colon, as well as mucosal tissue damage [1]. Although many studies suggest that genetic and environmental factors, infection, and immune system disorders are involved in the development of UC, its cause and underlying mechanisms remain unclear [2]. Several animal models of experimental colitis have been developed to help investigate the molecular and cellular mechanisms of the disease. Among these, the dextran sodium sulfate- (DSS-) induced experimental colitis model is one of the best studied. It is initiated by DSS-induced damage to the intestinal epithelial cells but is dependent on the presence of both commensal microflora [3, 4]. Similar to human IBD, DSS-induced colitis is limited to the colonic mucosa and is characterized by diarrhea, bloody feces, weight loss, colonic ulceration, and a histopathological picture of inflammation, consisting mainly of infiltrating macrophages and granulocytes [3, 4].

NF-*κ*B has represented a paradigm for signal transduction and gene regulation implicated in numerous diseases including malignancies and chronic inflammatory disorders [5, 6]. NF-*κ*B is normally sequestered in the cytoplasm by a family of inhibitory proteins known as inhibitors of NF-*κ*B (I*κ*Bs). Upon activation, I*κ*B is phosphorylated by the I*κ*B kinase (IKK) complex, and the phosphorylation of I*κ*B results in its degradation [7]. Subsequently, NF-*κ*B is translocated to the nucleus and initiates downstream target genes transcription related to inflammation and immunity [6]. It has been reported that NF-*κ*B plays a critical role at IBD onset in Crohn's disease (CD) and UC patients as well as experimental colitis models [8, 9].

Recently, pathogenic mechanism of UC is considered to be dysregulation of the intestinal immune response to intestinal environmental antigens, such as gut microbiota [10, 11]. Lipopolysaccharide (LPS), major constituent of outer membranes of Gram-negative bacteria, is a pivotal risk factor and prominent stimulus for inflammatory mediators releasing [12]. LPS would trigger the innate immune systems by activating Toll-like receptors (TLRs) superfamily in innate immune cells such as dendritic cells and macrophages [13, 14]. Among TLRs, TLR4 is a major LPS receptor and mediates the most of inflammation responses to LPS by activating NF-*κ*B protein, via myeloid differentiation factor 88- (MyD88-) dependent and MyD88-independent pathway [13–15]. Then, the activated NF-*κ*B induces the productions of proinflammatory cytokines such as tumor necrosis factor-*α* (TNF*α*), interleukin-1*β* (IL-1*β*), and IL-6 [14–16], resulting in severe intestinal injury and formation of UC. Alteration in cytokines production by macrophages is one major component of the pathology of UC. Therefore, strategies for targeting inflammatory macrophages and focusing on downregulating the TLR4/NF-*κ*B signaling pathways inflammatory responses would provide potential therapeutic effects for UC.

Recent advances in large-scale RNA sequencing (RNA-seq) have led to the identification of novel RNA species encoded in the genome, many of which are long noncoding RNAs (lncRNAs) [17, 18]. lncRNAs are arbitrarily defined as having ≥200 nt, which discriminates them from small noncoding RNAs. lncRNAs have emerged as important regulators of gene expression, with an accumulating body of evidence linking lncRNAs to a plethora of human pathologies, including inflammatory diseases [19]. Meanwhile, the role of lncRNAs in intestinal diseases remains poorly understood.

Irritable bowel syndrome (IBS) is characterized by abdominal pain or discomfort associated with altered bowel function, such as constipation or diarrhea, without significant structural changes or inflammation [20]. IBD is characterized by abundant inflammation in the bowel which can result in symptoms such as abdominal pain, cramping, diarrhea, and bloody stools. Approximately 60–80% of patients with IBD suffer from irritable IBS-like symptoms such as increased abdominal pain, bloating, distention, and stool alteration [21, 22].

Traditional Chinese medicine has been widely used in many kinds of diseases and has shown excellent curative effect [23, 24]. Guchang capsule (GC) is a Chinese materia medica standardized product extracted from 15 Chinese traditional medical herbs and is widely used for treatment of patients with irritable bowel syndrome (IBS) in clinical setting. However, the function of GC in IBD and the molecular mechanisms still remain unclear. Because of the overlapping symptom of IBS and IBD, we hypothesized if GC could have beneficial effect on IBD. In this paper, we described the effect of GC in DSS-induced experimental colitis which extended the research of GC in intestinal disease and explored the underlying mechanisms which may be associated with the production of inflammation related lncRNAs.

## 2. Materials and Methods

### 2.1. Vegetal Materia and Reagents

Guchang capsule (GC) consists of Halloysite,* Coptis*,* Phellodendron amurense*, Myrobalan, Nutmeg,* Cortex Magnoliae officinalis*, Fructus Evodiae, Qu Jian, Cinnamon, Rhizoma Zingiberis, Chinese Prickly Ash,* Ligusticum wallichii*, Concha Ostreae, Gallnut, and Dark Plum Fruit (Zhongxin Pharmaceutical Corporation, Tianjin, China). We dissolved the drug powder in sterile water at 0.1 g/mL GC for the further study:* in vivo*, GC were given intragastrically (2 g/kg);* in vitro*, GC were added into culture medium at a final concentration of 1 mg/mL. LPS (*E. coli*: Serotype O55:B5) and phorbol 12-myristate 13-acetate (PMA) were purchased from Sigma-Aldrich (St. Louis, MO, USA) and used at a final concentration of 100 ng/mL. Dextran sulfate sodium (DSS, molecular weight 36–50 kDa) was obtained from MP Biomedicals Inc. (Irvine, CA, USA).

### 2.2. Mice and Cells

Male C57BL/6J mice weighing 20–25 g (aged 8–10 weeks) were used in this study; all mice were obtained from Beijing Vital River Laboratory Animal Technology Co., Ltd. (Beijing, China). The animals were fed a standard diet and water was provided ad libitum. The mice were kept at room temperature (controlled at 25°C) with a light-dark cycle of 12 h each day. The experiments were carried out according to the National Institutes of Health Guide for the Care and Use of Laboratory Animals approved by the Animal Ethics Committee of the Scientific Investigation Board of Tianjin Medical University. Mouse macrophage cell line RAW264.7 and human monocytic leukemia cell line THP-1 were obtained from American Type Culture Collection (Manassas, VA). The cells were cultured at 37°C under 5% CO_2_ in DMEM supplemented with 10% FBS (Invitrogen, Life Technologies), 100 U/mL penicillin, and 100 mg/mL streptomycin. Differentiation of THP-1 monocytes to human macrophages was induced by 0.5 mM phorbol 12-myristate 13-acetate (PMA) for 3 h; the differentiated cells were washed three times with PBS, followed by the further experiments.

### 2.3. Induction and Evaluation of DSS-Induced Colitis

The mice were randomly assigned to control, GC, control + DSS-treated, and GC + DSS-treated groups. Experimental colitis was induced by adding DSS to the drinking water to a final concentration of 4% (w/v) for 6 days; subsequently, mice were sacrificed at day 6 to separate the colons for tissue experiments or switched to regular drinking water until 15 days for survival data monitoring. The animals were given free access to water containing DSS for 6 days. Control animals received water without DSS. GC were given intragastrically once per day for 6 days from the first day, respectively. Mice were examined daily to determine their clinical disease activity index (DAI), which was based on the degree of body weight loss, stool consistency, and fecal blood (ranging from 0 to 12). Briefly, DAI was scored as follows: weight loss (no change = 0; <5% = 1; 6–10% = 2; 11–20% = 3; >20% = 4), stool (normal = 0; soft, well-formed = 1; soft without pellets = 2; diarrhea = 4), and blood (no blood = 0; visible blood in rectum = 1; gross bleeding in rectum = 2; visible blood on fur = 4). For histological analysis, the distal colonic specimens were fixed in 10% buffered formalin and embedded in paraffin. Sections were stained with H&E, and pathological scores, ranging from 0 to 6 (combining inflammatory cell infiltration score and tissue damage score), were determined as follows: inflammatory cell infiltration in the lamina propria (occasional inflammatory cells = 0; increased inflammatory cells = 1; confluence of inflammatory cells extending to the submucosa = 2; transmural extension = 3) and tissue damage (no mucosal damage = 0; lymphoepithelial lesions = 1; surface mucosal erosion = 2; extensive mucosal damage and extension into deeper structures of the bowel wall = 3).

### 2.4. ELISA

Distal parts of colons were homogenized in CelLytic buffer (Sigma-Aldrich, St. Louis, MO) supplemented with complete protease inhibitor mixture (Roche, Indianapolis, IN). Protein concentration was measured by BCA assay (Pierce, Rockford, USA). Cytokine content was determined as pg/mg of total colon protein. Antibodies, which include purified and biotinylated rat anti-mouse TNF*α*, IL-6, and IL-1*β*, were purchased from eBioscience and BD Biosciences Pharmingen. Quantitative ELISA was performed according to the manufacturer's instructions.

### 2.5. RNA Isolation and Quantitative PCR Analysis

The tissue samples were frozen and mechanically dissociated in TRIzol reagent (Invitrogen, Carlsbad, CA). Total RNA was extracted with TRIzol reagent according to the manufacturer's instructions. A LightCycler (ABI PRISM 7000; Applied Biosciences) and a SYBR RT-PCR kit (Takara Biotechnology, Dalian, China) were used for real time PCR analysis. GAPDH was used as the internal control, and 2^−ΔΔCT^ method was used to evaluate the relative quantities of each amplified product in the samples. For each qPCR analysis, three technical replicates were performed. Primer sequences used in quantitative PCR were shown in Table 1.

### 2.6. Western Blot Analysis

The cells were harvested after stimulation and washed three times by ice-cold PBS (MDL biotech, Beijing, China) on ice, and the cells were lysed with RIPA buffer (MDL biotech, Beijing, China) with proteasome inhibitor cocktail (Roche, Indianapolis, IN), followed by sonication. After centrifugation for 15 min at 13,000 g, supernatants were collected and the protein concentration of lysates was measured using Bio-Rad quantification assay (Bio-Rad Laboratories, Hercules, CA). Twenty micrograms of protein sample was incubated with loading buffer (Beyotime, Beijing, China) at 95°C for 10 minutes. Proteins were separated using 10% SDS-PAGE and transferred to a PVDF membrane (Millipore, Billerica, USA). The membrane was then blocked with 2.5% nonfat dry milk for 1 h. The antibodies specific for p65, phospho-p65, I*κ*B*α*, phospho-I*κ*B*α*, IKK*β*, phospho-IKK*β* (Cell Signaling Technology Inc., Beverly, USA), and *β*-Actin (Santa Cruz Biotechnology, Santa Cruz, USA) were added and incubated overnight at 4°C. After incubation with the corresponding horseradish peroxidase-conjugated secondary antibody (Santa Cruz Biotechnology, Santa Cruz, USA), the target protein was visualized by enhanced chemiluminescence (Thermo Fisher Scientific, Bremen, Germany).

### 2.7. Assay of Luciferase Reporter Gene Expression

RAW264.7 macrophages were cotransfected with NF-*κ*B luciferase reporter plasmid and pRL-TK-Renilla-luciferase plasmid using Jet-PEI transfection reagent (Polyplus, Illkirch, France) as previously reported [25]. 24 hours after transfection, the cells were left untreated or treated with LPS, with 1 mg/mL GC or sterile water coapplied into culture medium for 4 hours. The cells were harvested and washed by ice-cold PBS for three times and luciferase activities were measured with Dual-Luciferase Reporter Assay System (Promega, Madison, USA), according to the manufacturer's instructions. Data are normalized for transfection efficiency by dividing Firefly luciferase activity with that of Renilla luciferase. NF-*κ*B luciferase reporter plasmid and pRL-TK-Renilla-luciferase plasmid were gifts of X. Cao (Second Military Medical University, Shanghai, China).

### 2.8. Statistical Analysis

All data are presented as mean ± SD of three independent experiments. Statistical significance was determined with the two-tailed Student's *t*-test to compare two groups. One-way ANOVA was performed to compare three or more groups. If the ANOVA analysis was significant, the Newman-Keuls test was applied for comparison between each two groups. The survival curves were plotted according to the Kaplan-Meier method and compared using the log-rank test. A *p* value of less than 0.05 was considered statistically significant.

## 3. Results

### 3.1. Effect of GC on Body Weight, DAI, and Survival Rate in DSS-Treated Mice

**Figure 1:**
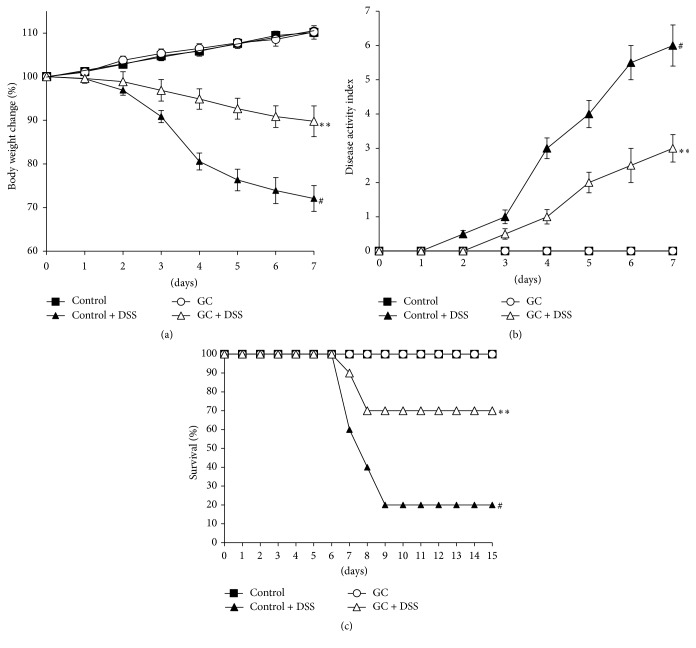
Effect of GC on body weight, DAI, and survival rate in DSS-treated mice. (a) Body weight change; (b) disease activity index; and (c) survival rate. Experimental model of ulcerative colitis was induced by administration of 4% dextran sulfate sodium (DSS) for 6 days and then switched to regular drinking water for data monitoring. GC were given intragastrically once per day for 6 days from the first day, respectively. Body weight change (a) and disease progression (b) were monitored daily from day 1 to day 7. Survival rate (c) was monitored until day 15 after the initiation of DSS. The data are representative of three independent experiments (*n* = 5 per group in (a-b) and *n* = 10 per group in (c), mean ± SD). ^*∗∗*^ *p* < 0.01 versus control + DSS group; ^#^ *p* < 0.05 versus control group.

To determine the potential role of Guchang capsule (GC) in experimental ulcerative colitis, mice were given 4% DSS for 6 days to induce acute colitis. The consumption of food and water was measured throughout the experiment, and there were no significant differences between the groups (data not shown). As shown in Figure 1(a), DSS-treated colitis mice exhibited profound body weight loss, whereas GC administration could significantly attenuate the loss of body weight. The clinical manifestation of the disease, as reflected by disease activity index (DAI), was markedly higher in the DSS-treated group compared with nontreated control group, but the treatment with GC significantly reduced the clinical scores of the DSS-induced colitis in mice (Figure 1(b)). Furthermore, the mortality of DSS-treated mice was improved by about 50% after GC administration (Figure 1(c)).

### 3.2. Effect of GC on Histology of Colon in DSS-Treated Mice

**Figure 2:**
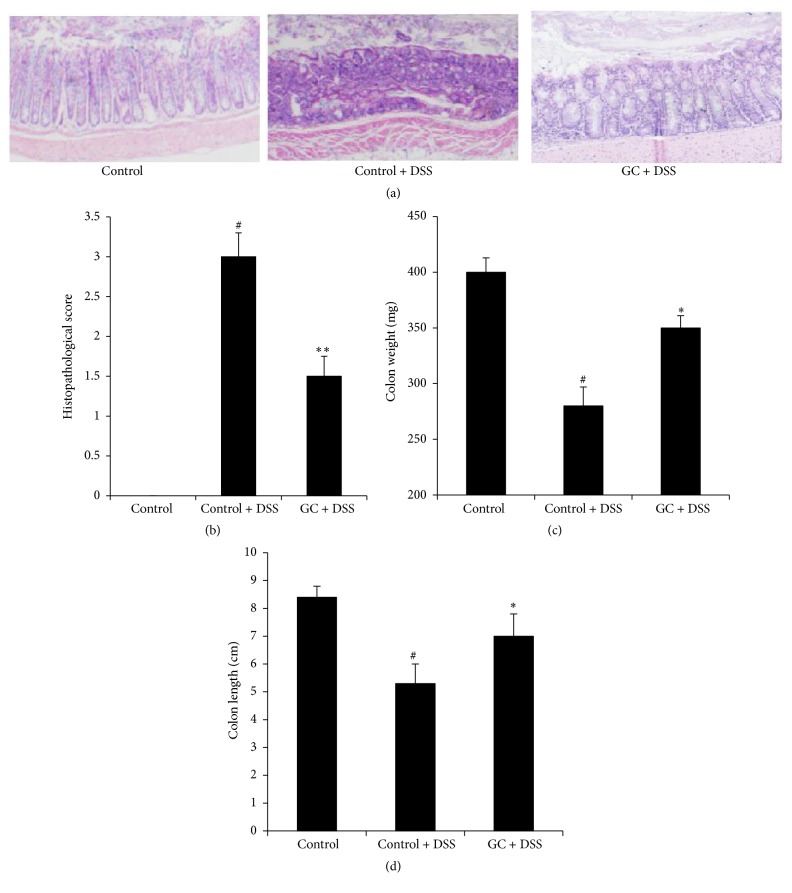
Effect of GC on histology of colon in DSS-treated mice. (a) Hematoxylin and eosin- (H&E-) stained sections of colons (original magnification ×100); (b) histopathological score; (c) colon weight; (d) colon length. Colitis was induced by administration of 4% DSS for 6 days. GC were given intragastrically once per day for 6 days from the first day, respectively. The mice were sacrificed at day 6 and colons were excised for indicated experiments. The data are representative of three independent experiments (*n* = 5 per group, mean ± SD). ^*∗*^ *p* < 0.05 versus control + DSS group, ^*∗∗*^ *p* < 0.01 versus control + DSS group, and ^#^ *p* < 0.05 versus control group.

Histological examination was also performed to validate the clinical data. DSS treatment induced significant histopathological changes in the colons of control mice that were characterized by massive inflammatory infiltrates and disruption of mucosal structures (Figure 2(a)). However, GC treated mice displayed less severe injury compared with control mice after DSS feeding (Figure 2(a)). The histopathological score of GC treated mice was significantly lower than that of control mice after DSS treatment (Figure 2(b)). After DSS-induced injury, the colon weight of GC treated mice was heavier than that of control mice (Figure 2(d)), and GC treated mice had longer colon length compared to control group after DSS feeding.

### 3.3. GC Administration Decreased Proinflammatory Cytokines Production in DSS-Treated Mice

**Figure 3:**
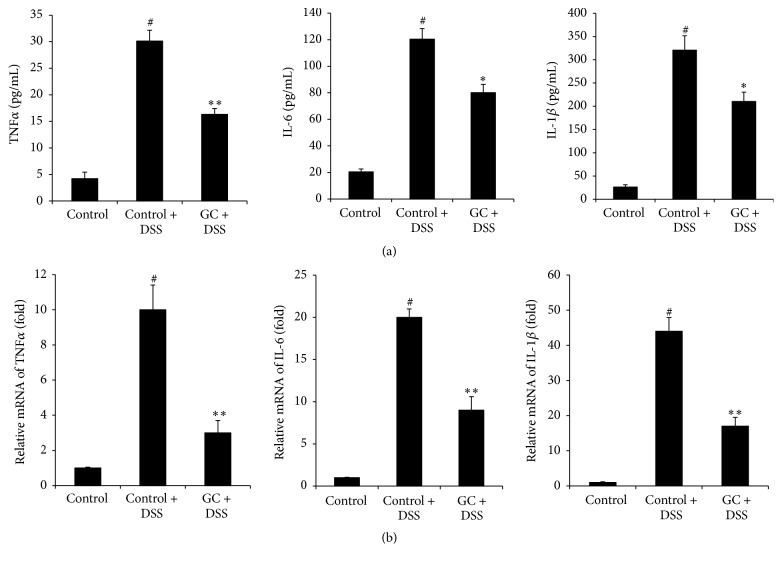
GC administration decreased proinflammatory cytokines production in DSS-treated mice. (a) ELISA analysis of secreted proinflammatory cytokines; (b) qPCR analysis of mRNA level of proinflammatory cytokines. Colitis was induced by administration of 4% DSS for 6 d. GC were given intragastrically once per day for 6 days from the first day, respectively. The mice were sacrificed at day 6. The protein level (a) or mRNA level (b) of TNF*α*, IL-6, and IL-1*β* in distal part of colons was analyzed. The data are representative of three independent experiments (*n* = 5 per group, mean ± SD). ^*∗*^ *p* < 0.05 versus control + DSS group, ^*∗∗*^ *p* < 0.01 versus control + DSS group, and ^#^ *p* < 0.05 versus control group.

To evaluate whether the protection from colitis induced by DSS in mice with GC treatment was associated with a reduction in the production of proinflammatory cytokines, after DSS challenge, expression levels of TNF*α*, IL-1*β*, and IL-6 in colons of control or GC treated mice were detected using ELISA (Figure 3(a)) and qPCR (Figure 3(b)). Significantly, both the mRNA and protein levels of proinflammatory cytokine were decreased in GC treated group.

### 3.4. GC Treatment Decreased Proinflammatory Cytokines Production in LPS Stimulated RAW264.7 Cells

**Figure 4:**
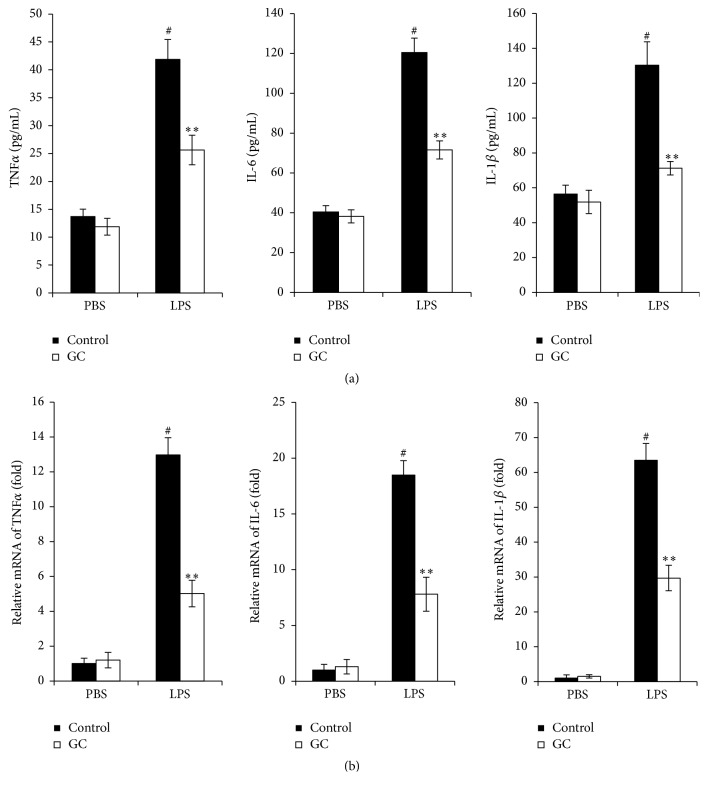
GC treatment decreased proinflammatory cytokines production in LPS stimulated RAW264.7 cells. (a) ELISA analysis of secreted proinflammatory cytokines; (b) qPCR analysis of mRNA level of proinflammatory cytokines. RAW264.7 cells were stimulated with LPS (100 ng/mL) together with GC (1 mg/mL) for 4 hours and the protein level (a) or mRNA level (b) of TNF*α*, IL-6, and IL-1*β* was analyzed. The data are representative of three independent experiments (mean ± SD). ^*∗∗*^ *p* < 0.01 versus control + DSS group; ^#^ *p* < 0.05 versus PBS + control group.

During intestinal mucosa damage, LPS secreted by the intestinal flora will stimulate infiltration of macrophages to activate the production of inflammatory factors, so we examined the effect of GC on LPS stimulated proinflammatory cytokines production in RAW264.7 cells. The same as the above results, the anti-inflammatory activity of GC was also confirmed in LPS stimulated RAW264.7 cells* in vitro*. Both the mRNA and protein levels of proinflammatory cytokines were decreased in GC treated group compared with control group after LPS stimulation (Figures 4(a) and 4(b)).

### 3.5. GC Suppressed NF-*κ*B Activation after LPS Stimulation in RAW264.7 Cells

**Figure 5:**
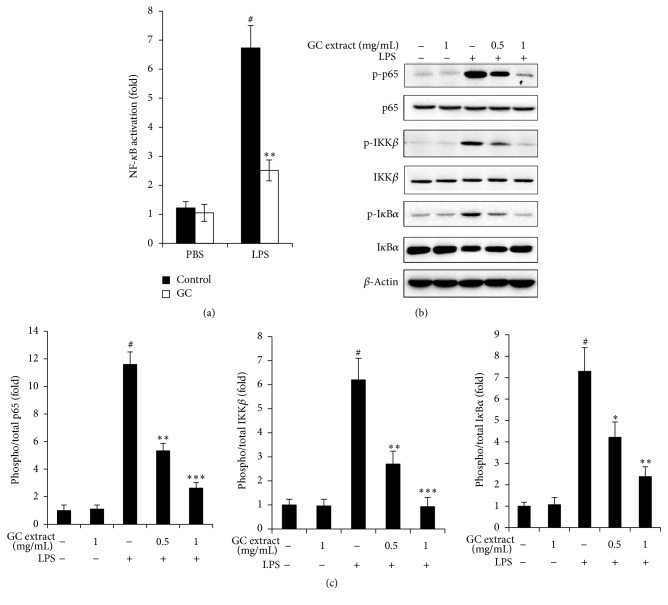
GC suppressed NF-*κ*B activation after LPS stimulation in RAW264.7 cells. (a) Luciferase reporter assay; (b) Western blot analysis; (c) quantification of protein level. RAW264.7 cells were transfected with NF-*κ*B luciferase reporter plasmid together with pRL-TK-Renilla-luciferase plasmid (internal control); 24 hours later the cells were stimulated with LPS (100 ng/mL) together with GC (1 mg/mL) for 4 hours; the luciferase reporter assay was performed to examine the activation of NF-*κ*B promoter (a); ^*∗∗*^ *p* < 0.01 versus control + DSS group; ^#^ *p* < 0.05 versus PBS + control group. The expressions of p-p65, p65, p-IKK*β*, IKK*β*, p-I*κ*B*α*, and I*κ*B*α* were examined by WB in RAW264.7 cells after stimulation with LPS and treatment with different quality of GC (b). Quantification of protein level of p-p65, p-IKK*β*, p-I*κ*B*α* in (b) and (c); ^*∗*^ *p* < 0.05 versus control + LPS group, ^*∗∗*^ *p* < 0.01 versus control + LPS group, ^*∗∗∗*^ *p* < 0.001 versus control + LPS group, and ^#^ *p* < 0.05 versus untreated control group. The data are representative of three independent experiments (mean ± SD in (a, c)).

Production of proinflammatory cytokines upon LPS depends mainly on the NF-*κ*B activation, so we hypothesized if GC would affect NF-*κ*B activation. To determine the function of GC on NF-*κ*B activation, first we transfected NF-*κ*B luciferase reporter plasmid into RAW264.7 cells; 24 hours later we stimulated cells with LPS together with GC or PBS treatment for 4 hours and measured luciferase activity. LPS-induced NF-*κ*B activation was greatly decreased by GC treatment compared with control group (Figure 5(a)). Western blotting showed that LPS-induced phosphorylation of NF-*κ*B/p65 and IKK*β* and I*κ*B*α* phosphorylation were all suppressed by GC treatment (Figure 5(b)).

### 3.6. GC Regulated NF-*κ*B Related lncRNAs Expression

**Figure 6:**
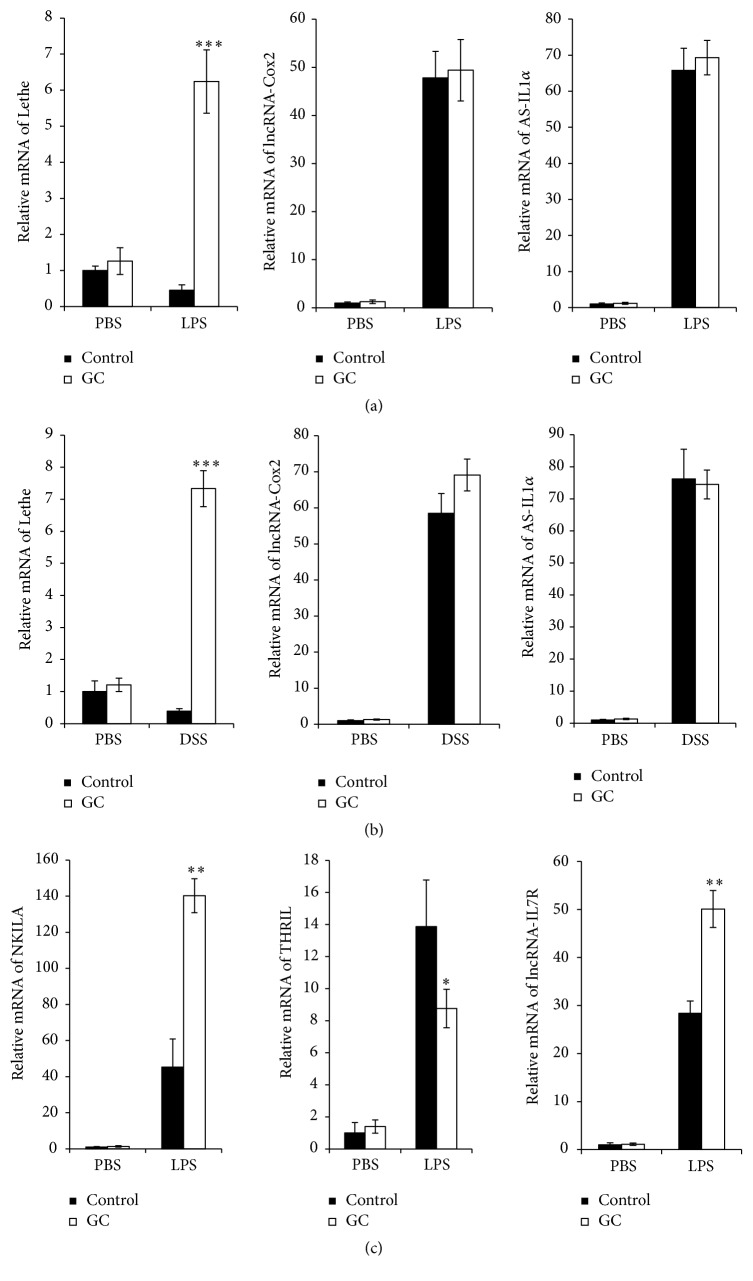
GC regulated NF-*κ*B related lncRNAs expression. (a) QPCR analysis of lncRNA Lethe, lncRNA-Cox2, and lncRNA AS-IL1*α* expression in RAW264.7 cells after LPS (100 ng/mL) and GC (1 mg/mL) treatment for 4 hours; (b) qPCR analysis of lncRNA Lethe, lncRNA-Cox2, and lncRNA AS-IL1*α* mRNA level in mice after DSS (4%) and GC (2 g/kg) treatment for 6 days; (c) qPCR analysis of expression of lncRNA NKILA, lncRNA THRIL, and lncRNA-IL7R in THP-1-derived macrophages after LPS (100 ng/mL) and GC (1 mg/mL) treatment for 4 hours. The data are representative of three independent experiments (*n* = 5 per group in (b), mean ± SD). ^*∗*^ *p* < 0.05; ^*∗∗*^ *p* < 0.01; ^*∗∗∗*^ *p* < 0.001 versus control + LPS (DSS) group.

In recent years, lots of long noncoding RNAs (lncRNAs) have been identified in the mammalian genomes, many of which have been implicated in a range of developmental processes and diseases [18, 26–28]. Though most of lncRNAs have been primarily studied in the context of genomic imprinting, developmental process, and cancer, lncRNAs are now emerging as important regulators of both innate and adaptive immune responses [29]. We hypothesized that GC could regulate lncRNAs expression in DSS-induced colitis; then we examined the expression of lncRNAs which were reported in regulating activation of NF-*κ*B pathway. Interestingly, lncRNA Lethe, reported as a negative regulator of NF-*κ*B, was significantly upregulated in GC group after LPS stimulation (Figure 6(a)) or DSS treatment (Figure 6(b)), but lncRNA-Cox2 and lncRNA AS-IL1*α* expressions were not affected (Figures 6(a) and 6(b)). We also examined the effect of GC on expression of lncRNAs which were reported in human THP-1-derived macrophages after LPS stimulation; we found that expressions of the negative regulatory lncRNA NKILA and lncRNA-IL7R were both upregulated, and the expression of lncRNA THRIL which was reported to downregulate TNF*α* production was suppressed (Figure 6(c)). These results indicated GC could regulate multiple NF-*κ*B related lncRNAs expressions in murine and human macrophage.

## 4. Discussion

Guchang capsule has been widely used for treatment of patients with IBS in clinical setting in China; because of the overlapping symptom between IBS and IBD, we wonder if GC could affect IBD progressing and we investigated the effect of GC in murine experimental colitis and revealed the potential molecular mechanisms in current study. To the best of our knowledge, this study is the first to systematically demonstrate the function of GC in DSS-induced murine experimental colitis and reveal the special effect of GC on regulating inflammation associated lncRNA expression. Our results suggested that GC treatment could attenuate DSS-induced injury and suppressed proinflammatory cytokines production in DSS-treated mice or LPS stimulated RAW264.7 cells. Furthermore, we found that GC treatment would inhibit the activation of NF-*κ*B and GC had different regulating effect on several kinds of NF-*κ*B/inflammation associated lncRNA.

Ulcerative colitis (UC) is one of the two major forms of inflammatory bowel diseases (IBDs), which are chronic, relapsing, idiopathic, and inflammatory conditions that are immunologically mediated. Diarrhea, abdominal pain, and bloody mucopurulent stool are the main clinical symptoms for UC [1, 2]. Currently, there are various evolving therapeutic options for UC. Immunosuppressive drugs such as TNF*α* antibody [30], azathioprine (AZA), and methotrexate (MTX) [31] have been adopted to control the symptoms. However, these immunosuppressants have their limitations in efficacy and safety [32, 33]. Therefore, novel strategies with high efficacy and safety are urgently required.

Establishment of UC by oral administration of DSS in the mouse is a widely used model for studies on this disease because this model resembles human UC and the resulting pathological conditions correspond very well to human. Acute ulcerative colitis induced by DSS showed intestinal bacterial infection and was characterized by massive infiltration of proinflammatory cells, such as macrophages, neutrophils, and CD4+ T cells, within the colonic walls, which destroy epithelium and shorten the colon length [34]. These infiltrated inflammatory cells are major producers of inflammatory mediators, such as TNF*α*, IL-6, and IL-1*β*, which contribute to pathogenesis [4]. Activation of NF-*κ*B is thought of as a strong inducer of these proinflammatory cytokine expressions [35, 36] and has been proposed as a major culprit and therapeutic target for IBD. Recent studies showed that many traditional Chinese medicines showed protective effect on DSS-induced colitis in mice. Song et al. reported that black tea extract could prevent LPS-induced NF-*κ*B signaling and attenuates DSS-induced experimental colitis [37]; Li et al. showed that Calculus Bovis Sativus (CBS) had protective effect on DSS-induced colitis and CBS significantly downregulated the mRNA expression of TNF*α*, IL-1*β*, and IL-6 in the colon tissue [38]. Dave et al. found that mulberry fruit prevents LPS-induced NF-*κ*B/pERK/MAPK signals in macrophages and suppresses acute colitis and colorectal tumorigenesis in mice [39]. Similar to their data, we found that GC showed beneficial effect on DSS-induced colitis in mice, such as less body weight loss, improved survival rate, weaker pathological damage, and decreased production of proinflammatory cytokines compared to the control group exposed to DSS, and GC treatment also attenuated cytokines production and suppressed NF-*κ*B activation in RAW264.7 cells after LPS stimulation.

In recent years, tens of thousands of long noncoding RNAs (lncRNAs) have been identified in the mammalian genomes, many of which have been implicated in TLRs/NF-*κ*B pathways. lncRNA Lethe was found to interact with NF-*κ*B subunit RelA to inhibit RelA DNA binding and target gene activation; furthermore, Lethe level decreases with organismal age, a physiological state associated with increased NF-*κ*B activity [19]. TLR signaling could induce lncRNA-Cox2, which serves as both repressor and activator of genes through interactions with various regulatory complexes [40]. A natural antisense transcript, AS-IL1*α*, was found to control inducible transcription of the proinflammatory cytokine IL-1*α* upon TLRs stimulations [41]. Liu et al. report that NKILA is an essential lncRNA that regulates NF-*κ*B signaling and represses inflammation; NKILA can bind to NF-*κ*B/I*κ*B complex and inhibits NF-*κ*B signaling by blocking the phosphorylation sites of I*κ*B and stabilizing the complex [42]. Li et al. identified that lncRNA THRIL was essential for induction of TNF*α* expression through its interaction with hnRNPL during innate activation of THP-1 macrophages [43]. Cui et al. identified lncRNA-IL7R which inhibited LPS-induced proinflammatory response through regulating trimethylation of histone H3 at K27 at the proximal promoters of inflammatory mediators [44]. We then examined the effect of GC on the expression of these lncRNAs in DSS-treated mice and LPS stimulated macrophages. Interestingly, we found that, in mouse macrophage cell line RAW264.7, lncRNA Lethe was decreased after DSS challenge or LPS stimulation in control group, while GC treatment significantly increased Lethe expression in these conditions. The expressions of lncRNA-Cox2 and AS-IL1*α* were not changed between GC group and control group after DSS treatment or LPS stimulation. We also detected the expression of inflammation associated lncRNAs such as NKILA, lncRNA THRIL, and lncRNA-IL7R which were discovered in human immune cells. In THP-1-derived macrophages, the expressions of NKILA and lncRNA-IL7R were significantly upregulated, and mRNA level of lncRNA THRIL which could inhibit TNF*α* expression was decreased. These findings suggested that GC could regulate diverse lncRNAs expressions; for example, in NF-*κ*B pathway and during proinflammatory cytokines production, GC could upregulate the negative regulatory lncRNAs expression; meanwhile it suppressed the expression of positive regulatory lncRNAs related to NF-*κ*B pathway, which resulted in ameliorate effect on ulcerative colitis.

## 5. Conclusions

In conclusion, our current study provided the evidence that Guchang capsule could ameliorate DSS-induced colitis and illustrated its potential mechanism by inhibiting NF-*κ*B activation through regulating expression of NF-*κ*B related lncRNAs. This study enriched the function of Guchang capsule in the treatment of ulcerative colitis and expanded our understanding of the mechanism of traditional Chinese medicine.

## Figures and Tables

**Table 1:** List of primers used in the study.

Number	Gene	Primer sequence	Primer length
1	TNF*α*	Forward 5′-GCCACCACGCTCTTCTGTCT-3′ Reverse 5′-TGAGGGTCTGGGCCATAGAAC-3′	20 bp 21 bp
2	IL-6	Forward 5′-ACAACCACGGCCTTCCCTAC-3′ Reverse 5′-CATTTCCACGATTTCCCAGA-3′	20 bp 20 bp
3	IL-1*β*	Forward 5′-ACCTTCCAGGATGAGGACATGA-3′ Reverse 5′-AACGTCACACACCAGCAGGTTA-3′	22 bp 22 bp
4	GAPDH	Forward 5′-ACCACAGTCCATGCCATCAC-3′ Reverse 5′-TCCACCACCCTGTTGCTGTA-3′	20 bp 20 bp
5	lncRNA Lethe	Forward 5′-CAGGACTGAGGAGGACATCA-3′ Reverse 5′-GCCTCTCTTCTCAGCGTTGT-3′	20 bp 20 bp
6	lncRNA-Cox2	Forward 5′-AAGGAAGCTTGGCGTTGTGA-3′ Reverse 5′-GAGAGGTGAGGAGTCTTATG-3′	20 bp 20 bp
7	lncRNA AS-IL1*α*	Forward 5′-AGCTCCCATGGATGCCTTTAG-3′ Reverse 5′-GCCCTAAGAACAGGGCTAGG-3′	21 bp 20 bp
8	lncRNA NKILA	Forward 5′-GAAACAGGTGCACGTTTCAGG-3′ Reverse 5′-ACAGATGTACCCTGGCAACC-3′	21 bp 20 bp
9	lncRNA THRIL	Forward 5′-AACTCCTGACCTCAGGTGATCCAT-3′ Reverse 5′-AAGGGAGTTTCAGAAGGTGTGGCT-3′	24 bp 24 bp
10	lncRNA-IL7R	Forward 5′-CCAGCCTTTGCCTCTTCCTTCAAT-3′ Reverse 5′-CCGTACCAAGTCTCTTAGCCCCTC-3′	24 bp 23 bp
